# Long-term potentiation in an innexin-based electrical synapse

**DOI:** 10.1038/s41598-018-30966-w

**Published:** 2018-08-22

**Authors:** Georg Welzel, Stefan Schuster

**Affiliations:** Department of Animal Physiology, University of Bayreuth, 95440 Bayreuth, Germany

## Abstract

Electrical synapses are formed by two unrelated gap junction protein families, the primordial innexins (invertebrates) or the connexins (vertebrates). Although molecularly different, innexin- and connexin-based electrical synapses are strikingly similar in their membrane topology. However, it remains unclear if this similarity extends also to more sophisticated functions such as long-term potentiation which is only known in connexin-based synapses. Here we show that this capacity is not unique to connexin-based synapses. Using a method that allowed us to quantitatively measure gap-junction conductance we provide the first and unequivocal evidence of long-term potentiation in an innexin-based electrical synapse. Our findings suggest that long-term potentiation is a property that has likely existed already in ancestral gap junctions. They therefore could provide a highly potent system to dissect shared molecular mechanisms of electrical synapse plasticity.

## Introduction

Electrical synapses formed by gap junction channels enable the direct spread of electrical current between coupled cells in vertebrates and invertebrates^1^. Two unrelated protein families constitute the gap junctions. While invertebrates use the primordial innexins, vertebrates use the modern connexins^2^. Although connexins and innexins share little sequence homology^2,3^, they form junctions with strikingly similar topology^4^. Vertebrate and invertebrate electrical synapses play crucial roles in the mechanisms of neuronal synchronization and excitability^5,6^, neuronal development^7,8^, lateral excitation in sensory systems^9–11^ or fast escape mechanisms^12,13^. In addition, basic functional features of connexin- and innexin-based electrical synapses are remarkably similar. They are both known to be regulated by pH^14^, Calcium, transmembrane voltage or transjunctional voltage^15–18^ and respond to neuromodulators^19–30^. However, in recent years it became increasingly evident that vertebrate connexin-based electrical synapses are capable of sophisticated forms of activity-dependent plasticity such as long-term potentiation^21,29–31^ or long-term depression^22,28,30,32,33^. Up to now, comparable data are still lacking for innexin-based electrical synapses^19^, suggesting that such higher forms of plasticity might constitute a functional difference between connexin- and innexin-based electrical synapses and that this difference was critical in the evolution of the modern connexins. Therefore, we studied an innexin-based electrical synapse in an invertebrate preparation to identify conditions under which it might be similarly capable of long-term plasticity. We show that it is possible to directly measure the gap junctional currents between the electrically coupled Retzius (R) cells in the nervous system of the leech (*Hirudo medicinalis*) and provide what seems to be the first direct demonstration of activity-dependent gap junction plasticity in an innexin-based electrical synapse.

## Results

### Direct measurement of gap junctional currents

**Figure 1:**
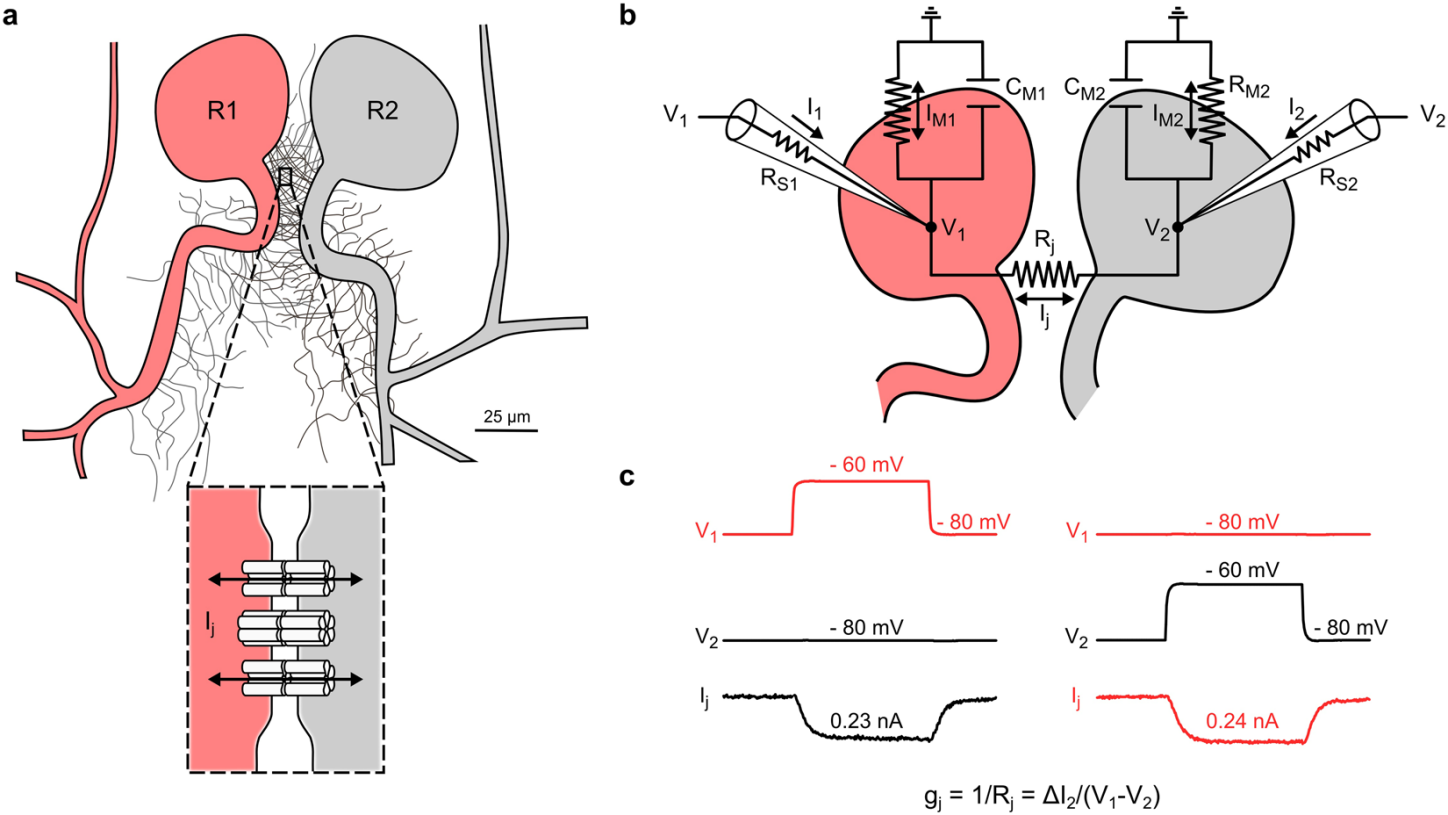
Direct measurement of gap junctional currents between electrically coupled Retzius cells. (**a**) Schematic representation of the somata and the neurites emerging from the primary axons of a pair of Retzius neurons (R1 and R2) in the leech nervous system (adapted from García-Pérez^34^). The somata are electrically coupled by non-rectifying electrical synapses (see inset) between pairs of neurites within the neuropilar arborizations proximal to the soma^34^, where application of voltage-clamp protocols was directly confirmed (Fig. S1). (**b**) Equivalent circuit of the electrically coupled R cells with recording microelectrodes (15–30 MΩ resistance). R_S1_, R_S2_: series resistances of microelectrodes. V_1_, V_2_: membrane potentials of R1 and R2. Cell membranes with resistances R_M1_, R_M2_ and capacities C_M1_, C_M2_. R_j_: gap junctional resistance. I_j_: gap junctional current. I_1_, I_2_: currents sent through microelectrodes. I_M1_, I_M2_: currents across membrane resistances. (**c**) To measure junctional currents, two coupled single-electrode voltage-clamp amplifiers clamped the cells at −80 mV. A probing brief depolarization to −60 mV applied to R1 is sufficient to detect the small junctional current (I_j_) from the change in current (∆I_2_) in the clamp circuit of the other cell, directly yielding junctional resistance (R_j_) and conductance (g_j_ = 1/R_j_).

To explore the extent to which innexin-based electrical synapses are capable of dynamic and activity-dependent plasticity, we chose to study the pair of Retzius (R) cells in the nervous systems of the leech (*Hirudo medicinalis*). The R cells are characterized by their large somata^34^ (60–80 µm) that are electrically coupled by non-rectifying electrical synapses^35^ between pairs of neurites in close proximity (<50 µm) to the soma^34^ (Fig. 1a). The strength of electrical coupling between neurons depends on gap junctional factors (gap junctional conductance) and non-junctional factors (passive and active neuronal properties)^19^. We intended to determine the strength of the electrical synapses and their possible changes as accurately as possible by using two synchronized discontinuous single-electrode voltage clamp amplifiers and dual whole-cell voltage-clamp recordings from the somata of R cell pairs. This technique, if applicable, allows a direct and precise measurement of the gap junctional currents (I_j_) without confounding effects of series resistances and of passive membrane properties that need to be kept in mind when using coupling coefficients^36^. Our approach is best understood with reference to an equivalent circuit (Fig. 1b) in which each R cell is represented by its membrane resistance and capacitance (R_M_, C_M_) and the electrical synapses between them are represented by a junctional resistance (R_j_). After directly confirming the applicability of our voltage-clamp protocols (Fig. S1) we could measure conductance (g_j_ = 1/R_j_) in the following way: First, we voltage-clamped both R cells at −80 mV, so that initially no current flowed. Then, we applied a brief small voltage jump to −60 mV to one of the two cells (called ‘R1’ in the following) so that the voltage drop at one hand caused a gap junctional current but at the other still did not cause the release of chemical transmitters. To maintain the small voltage step, the clamp circuit responsible for R1 must deliver an extra current that accounts for both the currents through the gap junctions (I_j_) and the current that leaks through the membrane (I_M1_) (Fig. 1b). The circuit to clamp R2, however, must exclusively compensate the current inflow through the gap junctions (−I_j_) (Fig. 1c) and is thus independent of all other potentially confounding parameters shown in Fig. 1b. Our method therefore allowed us to directly measure the gap junctional currents between the R cells and to straightforwardly explore their possible use-dependent regulation without any need to block chemical neurotransmission.

### Variations in gap junctional currents and the existence of phosphorylation sites

**Figure 2:**
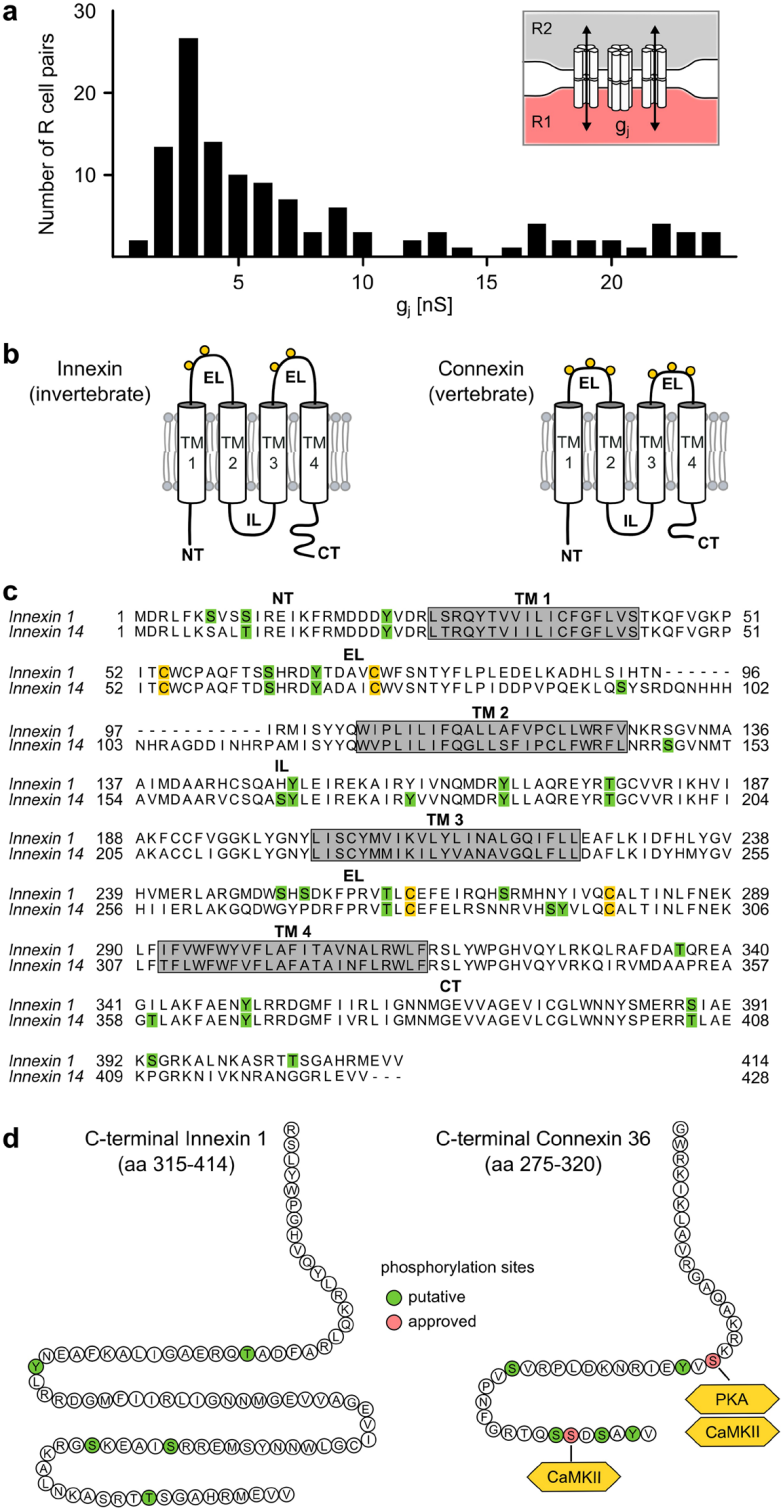
The structure and variable strength of Innexin-1 electrical synapses indicate their potential for plasticity. (**a**) The gap junctional conductances (g_j_) of electrically coupled R cells (see inset) show a wide distribution (n = 123 R cell pairs from 78 different animals) that differed significantly from a normal distribution (p < 0.001, Shapiro-Wilk). (**b**) Innexins and connexins share a common membrane topology with four transmembrane (TM), one intracellular (IL) and two extracellular (EL) domains^15^ each containing conserved cysteine residues (yellow circles) that are critical for stable gap junction channel formation^72^. Based on the sequence alignment and analysis in (**c**), we predicted the structure of Innexin-1 in comparison to the neuronal gap junction protein Connexin-36 of vertebrates. (**c**) Amino acid alignment of the two innexins that are predominantly expressed^38^ in our experimental system, the leech R cells. NetPhos 2.0^70^ was used to predict putative phosphorylation sites (green) and TMHMM 2.0^71^ was used to predict transmembrane domains (TM, grey boxes) of Innexin-1 and Innexin-14, respectively. Alignments were performed using Clustal W software. (**d**) Putative phosphorylation sites in the Innexin-1 carboxyl tail compared to the approved and putative phosphorylation sites in Connexin-36^42,45^.

Before embarking on a search for activity-dependent plasticity we first addressed two aspects. First, we checked the extent of variability in the conductance g_j_ of the innexin gap junctions across different R cell pairs. Our results clearly show that g_j_ does indeed vary within a large range and can differ by a factor of more than 20 (Fig. 2a). Because the variations of g_j_ would affect synchrony of spiking in the pair of R cells (Fig. S2c), they would have a potential functional significance, for instance, in the leech’s escape circuitry^37^ (Fig. S2a,b).

Second, we checked whether Innexin-1, the gap junction protein predominantly expressed in the R cells^38,39^, would potentially have phosphorylation sites that in connexin-based electrical synapses have been linked to the regulation of trafficking and turnover of gap junctions but also to changes in the open probability of existing gap junction channels^40^. In the case of Connexin-36, different phosphorylation associated pathways can directly regulate the strength of electrical coupling^30,41–49^ and some might be involved in activity dependent-plasticity of electrical synapses^50^. An analysis of the primary amino acid sequence of Innexin-1 identified putative phosphorylation sites in their carboxyl tail (Fig. 2c,d). It is important to stress that this does not imply any involvement in plasticity. However, it is a similarity that is, along with the same overall topology^4,15,51^ (Fig. 2b,c), shared by innexins and connexins and that could potentially be useful in plasticity. In summary, the variability (Fig. 2a), its potential behavioural significance (Fig. S2), and the occurrence of phosphorylation sites in its innexins suggests that the R cell pairs are an interesting system to probe for long-term plasticity in innexin-based synapses.

### Activity-dependent long-term potentiation of innexin-based gap junctions

**Figure 3:**
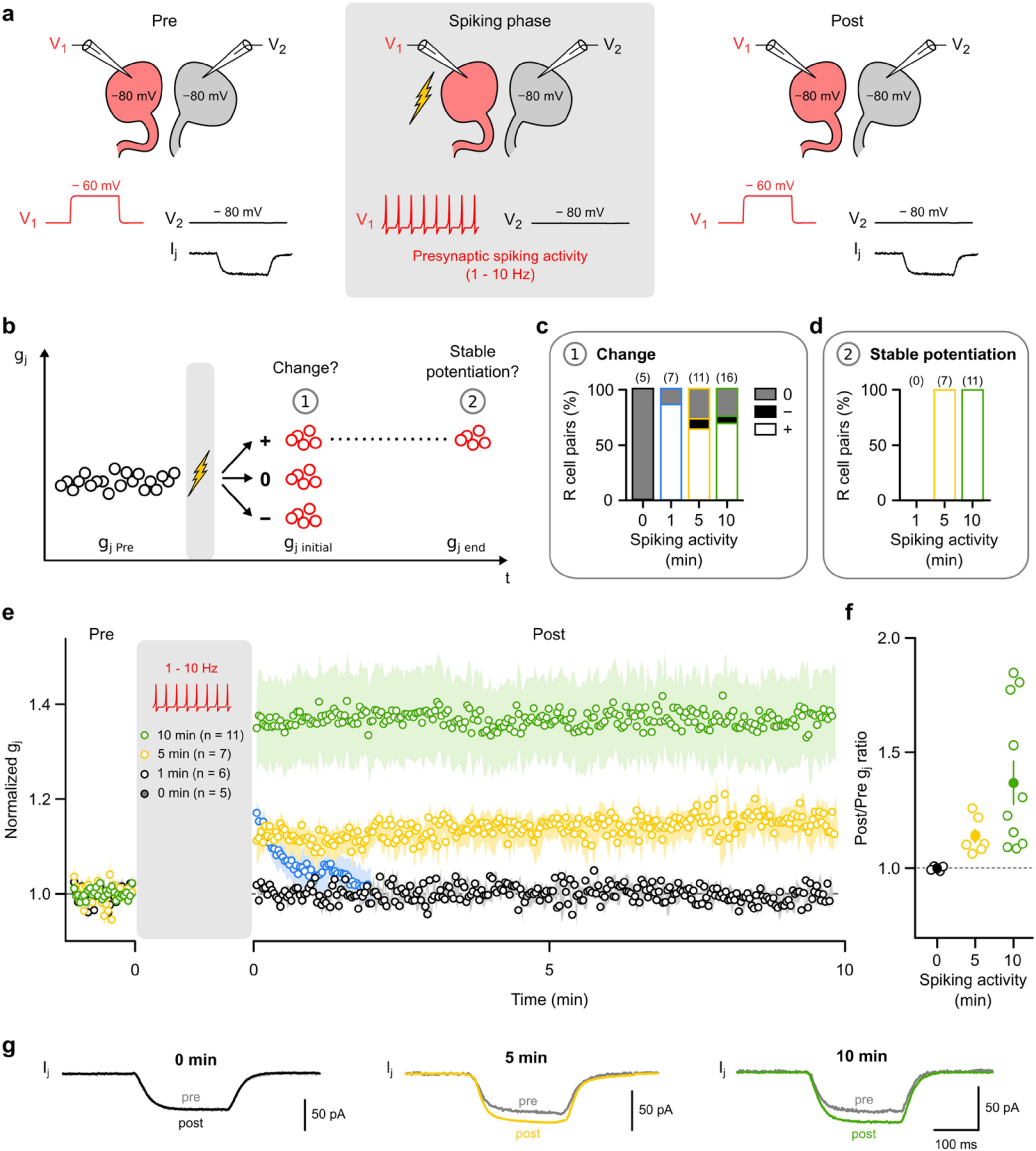
Innexin-based electrical synapses are capable of long-term potentiation (LTP). (**a**) Gap junctional conductance (g_j_) was determined every 2 s before (pre) and after (post) a spiking phase (flash symbol) during which current (2 nA, in current clamp mode) was injected into one cell of the pair (presynaptic) to cause spiking for 1, 5 or 10 min at a rate between 1 and 10 Hz. The other cell (postsynaptic) remained voltage-clamped at −80 mV (see Fig. S1). Additional controls (0 min) in which both cells remained voltage clamped throughout the experiment confirmed the stability of g_j_ in absence of the spiking phase. (**b**) After the spiking phase, the average g_j_ (g_j initial_) of the first 5 trials (i.e. the first 10 seconds) was used to form three categories: potentiation (+; g_j initial_ > mean g_j Pre_ + SD_Pre_), depression (−; g_j initial_ < mean g_j Pre_ − SD_Pre_) and no change (0). If a change had occurred, its stability was assayed using the average g_j_ of the last 5 trials of the 10 min observation period (g_j end_). To be considered stable, we required g_j end_ > g_j initial_ − SD_initial_. (**c**) Proportion of R cell pairs with increased (white), decreased (black) or unchanged (grey) g_j initial_ after the prior spiking phase of either 1, 5 or 10 min. (**d**) Proportion of R cell pairs in which an initial increase in g_j_ was stable, i.e. all of the recordings after 5 min and 10 min, but none after 1 min. (**e**) Normalized g_j_ (mean ± SEM) before and after the prior spiking phase (1, 5 or 10 min) averaged across all those R cell pairs shown in (**c**) that showed initial potentiation. Additionally, averaged controls without spiking activity (0 min) are shown to illustrate stability of g_j_ in absence of a spiking phase. (**f**) Ratio of the average g_j_ during the 10 min observation period (Post) and the respective average baseline g_j_ (Pre) for each R cell pair shown in (**e**). Mean values, averaged across the R cell pairs, are indicated by filled dots. (**g**) Average junctional currents (I_j_) in representative experiments before (pre) and after (post) 0 min (black), 5 min (yellow) or 10 min (green) of presynaptic spiking activity.

To directly test whether g_j_ can be modulated in an activity-dependent manner, we selectively induced spiking at average rates of 1–10 Hz in one cell of the pair (labelled the ‘presynaptic’ cell). This was achieved by releasing this cell from the voltage-clamp condition and injecting a prolonged depolarizing current (2 nA) for a set interval of time, either 1 min, 5 min or 10 min. Subsequently, the cell was voltage-clamped again and measurement of g_j_ proceeded as described. The second R cell of the pair (labelled ‘postsynaptic’) remained voltage-clamped at −80 mV all the time. Additional controls (0 min spiking activity in Fig. 3e) in which both cells remained voltage-clamped throughout the complete observation time confirm the stability of g_j_ (also see Fig. S3). For each R cell pair, we probed g_j_ before (baseline) and after the spiking phase (Fig. 3a). The protocol was chosen to be comparable to the hallmark experiments that demonstrated long-term plasticity in connexin-based electrical synapses^22,31–33,50,52^. The adequacy of the voltage clamp protocols was directly confirmed, also during presynaptic spiking, by monitoring the membrane potential in the postsynaptic cell with an additional independent microelectrode (Fig. S1).

In analysing the data we asked two questions: Firstly, whether the spiking phase caused any initial changes in g_j_ and, secondly, whether any of these initial changes would be stable (Fig. 3b). To be classified as an initial change, the mean g_j_ of the first 5 trials (g_j initial_) that followed directly in the first 10 seconds after the spiking phase had to differ by one standard deviation (SD) from the baseline, either upwards (>baseline + SD), or downwards (<baseline − SD) (Fig. 3b). Such changes occurred in 85.7% of the R cell pairs after the 1 min spiking phase, in 90.9% pairs after 5 min of spiking and in 93.8% pairs after 10 min of spiking. The proportions of these initial changes were not significantly dependent on the duration of the preceding spiking phase (P = 0.591, Chi2 test). In the controls (0 min of spiking), no change was detected, neither initially (Fig. 3c) nor later (Fig. S3). The next task was then, secondly, to test if any initial changes, when they occurred, would be stable. To do this, we first examined those R cell pairs with an initial increase in g_j_ and checked whether the increase was still present after our 10 min observation period (Fig. 3b). This was never the case in each of the R cell pairs (n = 6) that had undergone the brief 1 min spiking phase (Fig. 3d). However, after both 5 min and 10 min of prior spiking, the initial increase was stable in each of the R cell pairs (Figs 3d,e and S5) and the average g_j_ was significantly increased (after 5 min of spiking: 114.0% ± 0.1% of baseline, n = 7, p < 0.01; after 10 min of spiking: 136.9% ± 0.3% of baseline, n = 11, p < 0.001; one-tailed paired Wilcoxon test) (Fig. 3f). After both 5 and 10 min of spiking, one case was observed each with an initial decrease (Fig. 3b), one of them was stable (5 min). Even when this case was included as well as the cases in which no change had occurred, then the average g_j_ of the 10 min observation period was still significantly increased (after 5 and 10 min of spiking: p < 0.05, 0.01, respectively; two-tailed paired Wilcoxon test) (Fig. S4a).

We found no signs that the potentiation would start to decrease after the 10 min at which we decided to check for stability (Fig. S5). Additional recordings also showed that the increase in the synaptic strength was stable during 20 min of recording, again with no sign of a decline (Fig. S6). Without a spiking phase all R cell pairs showed stable g_j_ (Figs 3e and S3) demonstrating that the significant increase was not just a time-dependent run-up of g_j_ or confounded by large variations in the pre-induction levels of g_j_.

The degree of LTP did not depend on the pre-stimulation (baseline) level of g_j_ (r^2^ = 0.001, p = 0.970, Pearson correlation) and neither correlated with the total number of action potentials fired (r^2^ = 0.008, p = 0.564, Pearson correlation) nor with the average firing frequency (r^2^ = 0.044, p = 0.176, Pearson correlation) during the spiking phase (Fig. S7). When the spiking phase lasted only for 1 min, then subsequent changes in g_j_ were only short-lived (Fig. 3d,e). In addition, the level of potentiation was significantly higher after 10 min of spiking compared to 5 min of spiking (p < 0.05, unpaired one-tailed t-test). Thus, the duration of the spiking activity but not the firing frequency (above 1 Hz) or the number of action potentials seemed to be critical to induce LTP. In summary, these findings provide, to our knowledge, the first demonstration of activity-dependent LTP in any innexin-based electrical synapse.

### LTP is dependent on intracellular calcium

**Figure 4:**
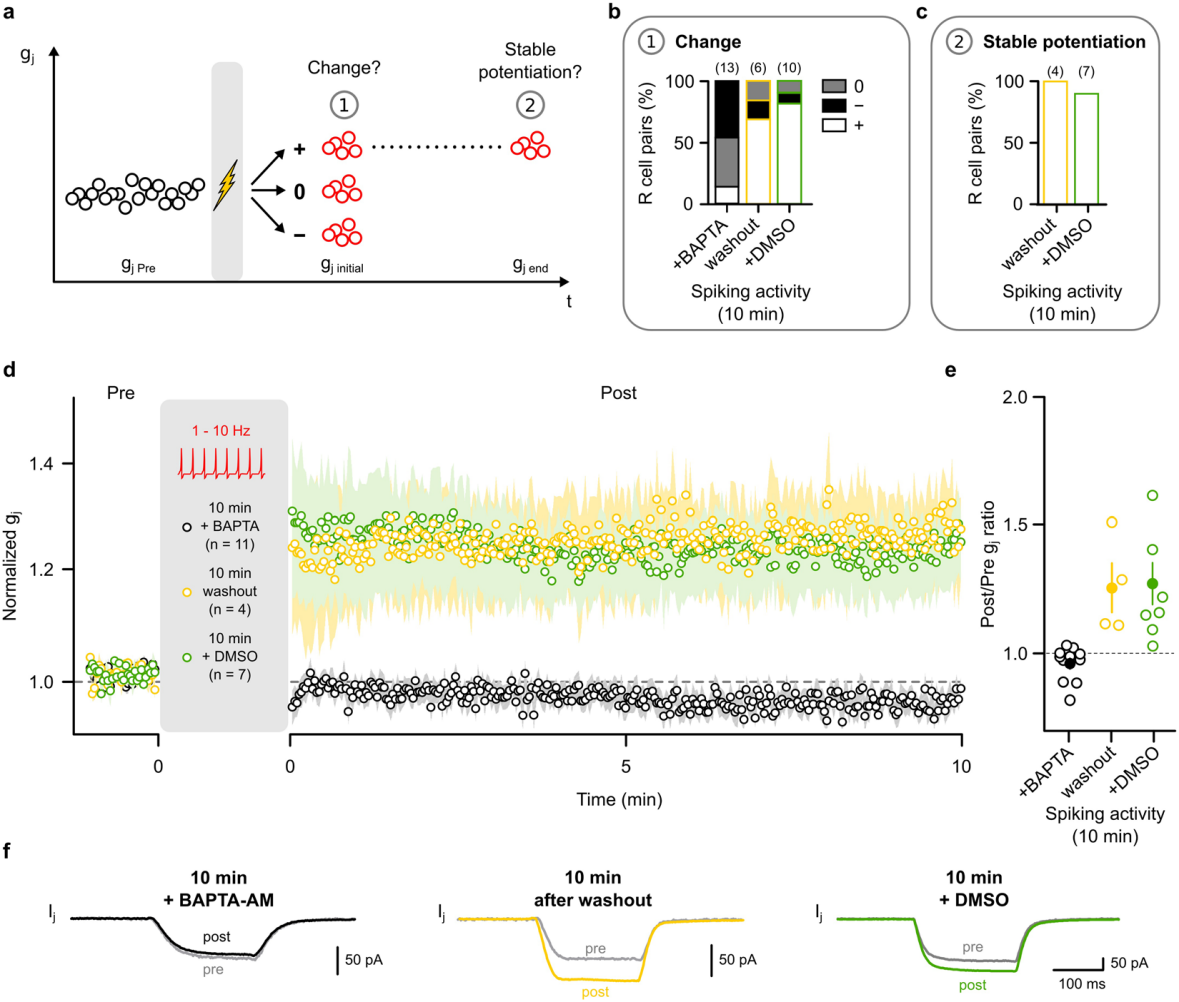
Activity-dependent LTP is dependent on intracellular calcium. (**a**) The average g_j_ of the first 5 trials (g_j initial_) after the spiking phase was determined to form three categories: potentiation (+; g_j initial_ > mean g_j Pre_ + SD_Pre_), depression (−; g_j initial_ < mean g_j Pre_ − SD_Pre_) and no change (0). The average g_j_ of the last 5 trials of the 10 min observation period (g_j end_) was determined to test if the initial change was stable (i.e. if g_j end_ > g_j initial_ − SD_initial_). (**b**) Proportion of R cell pairs with potentiated (white), depressed (black) or unchanged (grey) g_j initial_ subsequent to a 10 min period of spiking in presence of either BAPTA-AM in the external solution (50 µM in DMSO), the solvent alone and after washout of BAPTA-AM. (**c**) Proportion of R cell pairs in which an initial potentiation was stable. (**d**) Averaged normalized g_j_ (mean ± SEM) before and after spiking activity (10 min) in the majority of R cell pairs. In the presence of BAPTA-AM these were the ones with no change in g_j initial,_ after washout of BAPTA-AM or in presence of DMSO these were the ones with an initial increase of g_j_. (**e**) Ratio of the average g_j_ during the 10 min observation period (Post) and of g_j_ during the pre-spiking baseline phase (Pre) of each of the R cell pairs shown in (**d**). Averages across pairs are indicated by filled dots. (**f**) Average junctional currents (I_j_) in representative experiments before (pre) and after (post) 10 min spiking activity in presence of BAPTA-AM (black) in the external solution, its solvent (green) and after washout of BAPTA-AM (yellow).

The findings of Fig. 2d would be compatible with the view that LTP in the electrical synapse of the leech R cells could potentially be mediated by phosphorylation or dephosphorylation of Innexin-1. Phosphorylation-related modulation of Connexin-36-based electrical synapses often depends on calcium-dependent signalling pathways^22,45,47,48,50^. We therefore repeated the experiments in the presence of BAPTA-AM (in DMSO), a membrane permeable intracellular calcium chelator (Fig. 4). This indeed prevented the occurrence even of initial changes of g_j_ after a prior 10 min spiking phase in most R cell pairs (Fig. 4a,b). An increase was observed in only 15.4% of the cases, which is significantly lower than in experiments without BAPTA-AM (p < 0.01, Chi2 test). The predominant effects were no change (46.1%) or an initial decrease (38.5%) of g_j_. Figure 4d shows the time course of g_j_ across all pairs in which the 10 min spiking period had led to no change. The deviation from baseline was not significant (n = 6, p = 0.313, two-tailed paired Wilcoxon test; Fig. 4e). When all measurements were taken together (i.e. when the cases with an initial increase and decrease were also included) we also found no significant difference in the average g_j_ from baseline (p = 0.311, two-tailed paired Wilcoxon test) (Fig. S4b).

After subsequent washout of BAPTA-AM, we were again able to induce an increase in g_j initial_ in most of the R cell pairs (66.7%) after 10 min of prior spiking (Fig. 4a–c). In each of these cases, the increase in g_j initial_ was stable (Fig. 4c,d): When averaged 10 min after the initial increase g_j_ was 125.4% ± 0.1% of the baseline (n = 4, p < 0.05, one-tailed paired Wilcoxon test) (Fig. 3e). We note, however, that when the pairs in which an initial increase occurred were pooled with those with no change or a decrease, then there was no significant difference in the average g_j_ after 10 min (p = 0.625, two-tailed paired Wilcoxon test) (Fig. S4b).

In control experiments with only the solvent DMSO we were also able to induce increased values of g_j initial_ in most of the R cell pairs (80.0%) after 10 min of spiking (Fig. 4b) and in almost all of them (7 of 8) the increase was stable (Fig. 4c,d), with an average g_j_ 123.5% ± 0.1% of the baseline (n = 7, p < 0.01, one-tailed paired Wilcoxon test) (Fig. 4e). When the measurements with an initial increase were pooled with those with no change or a decrease, then the average g_j_, measured 10 min after the initial phase, was still significantly larger than the baseline level (p < 0.05, two-tailed paired Wilcoxon test; Fig. S4b). These results showed that it was BAPTA-AM and not the solvent that inhibited the induction of LTP (Fig. 4d,e,f). Hence, innexin-based electrical synapses use calcium-dependent regulatory mechanisms for long-term potentiation.

## Discussion

In this study, we unequivocally and for the first time demonstrate that innexin-based electrical synapses are capable of a sophisticated form of plasticity that can underlie learning and memory. We showed that dual whole-cell voltage-clamp recordings from the somata of electrically coupled Retzius cells in the leech can be used to directly examine activity-dependent changes of their electrical synapses. As recently emphasized^53^, this depended on the close proximity (<50 µm) of the electrical synapses to the soma^34^. By using two discontinuous single-electrode voltage clamp amplifiers we were thus able to experimentally measure the gap junctional currents without potentially confounding effects from instabilities or modulations of membrane resistance and series resistance^36,54^. Furthermore, we have confirmed that the membrane potential of the large soma of the R cells can be adequately controlled (Fig. S1) so that somatic voltage-clamp recordings can be used to accurately measure activity-dependent changes of gap junctional currents between R cell pairs. We thus provide a system that would be well suited for the future dissection of molecular mechanisms of electrical synapse plasticity and for probing which mechanisms might be shared by innexin- and connexin-based synapses.

Long-term potentiation is a fundamental mechanism of plasticity in vertebrates^55^. The discovery that long-term potentiation is not only seen in chemical synapses but also in electrical synapses is rather recent^55^ and its impact on brain function and dysfunction^55^ is not yet understood. In the leech, the adaptive significance of long-term potentiation of the electrical coupling of R cells could, for instance, be to adjust its life-saving withdrawal reflex. The coupled R cells are part of a neuronal circuit (Fig. S2a) that is necessary for modulating whole body shortening in response to threatening stimuli^37^. Within this circuit, the so-called S interneuron (S cell) plays a critical role for adapting the strength of the shortening reflex. The S cell excites the motor neurons responsible for the shortening contractions^56,57^. Its excitability and firing rate is regulated by the presynaptic release of serotonin by the R cells. The S cell itself feeds back onto the R cells, exciting them through a disynaptic pathway, thus creating a positive feedback loop^37,58^ (Fig. S2a). As the electrical synapses formed between the R cells have integrative functions and modulate the synchronous spiking (Fig. S2c) and excitability^59^ of R cells, the long-term plasticity of their gap junctions will have a direct impact on sensitization and dishabituation of the life-saving withdrawal reflex of the leech. We emphasize this to suggest why a capacity of long-term plasticity of electrical synapses (as suggested by Tsai *et al*.^60^) might have evolved early as an adaptive property of escape circuitry long before the advent of the chordates.

In vertebrates, it has been suggested that activity-dependent changes of electrical synapses could be driven by regulating the connexin biosynthesis and junctional plaque assembly and turnover^61,62^ or by changing the open probability of pre-existing gap junction channels^41,48^. Both mechanisms are assumed to occur at two different temporal levels^61–63^. While the regulation of open probability can take place within minutes, the rate of insertion or deletion of gap junction channels is changed on a time-scale of hours. Thus, our finding of an increase of g_j_ following 5 or 10 min of presynaptic spiking activity and subsequent voltage-clamping would seem to be more likely based on a regulation of the open probability of the channels during the spiking phase. Changing the open probability of connexins is supposed to be regulated by different signalling pathways that result in the phosphorylation or dephosphorylation of the cytoplasmic carboxyl tails of connexins^40,42,44,45^. One particularly interesting phosphorylation pathway is associated with the activation of NMDA receptors and the accompanying increase in the intracellular concentration of calcium. This in turn activates the calcium-calmodulin-dependent kinase II, which directly regulates the gap junctional conductance [for review, see^20^]. Although presently nothing is known about similar mechanisms in innexin-based electrical synapses, our results show that the induction of activity-dependent LTP in the leech also requires the availability of free intracellular calcium. Furthermore, R cells show a high expression of NMDA receptors^64^ and receive glutamatergic input from the S cells (*via* an interneuron)^37^. The firing activity of the S cells is regulated by the R cells itself^37,58^, which raises the possibility that spiking in one R cell during the phase in which one cell was released from voltage-clamping, could cause increased glutamatergic input and activation of NMDA receptors in the R cells.

Innexins are the primordial gap junction proteins and are already present in the simple neuronal nets of bilaterians^2^. Thus, it would be conceivable and fully in accord with our findings that the connexins that evolved much later in deuterostomes, exploit the same synaptic mechanisms of use-dependent plasticity that were already present in the primordial innexin-based electrical synapses.

In summary, our findings provide the first direct evidence that activity-dependent plasticity of electrical synapses is not restricted to the connexin-based gap junctions of vertebrates but is shared by innexin-based electrical synapses of invertebrates. This finding is important in two ways: first it raises the possibility that some mechanisms of long-term plasticity in electrical synapses and their role in learning and memory, might be shared by vertebrates and invertebrates. These could then be studied in an amenable invertebrate system, much as has been so important for our understanding of plasticity in chemical synapses^65–68^. Second, it suggests that a much deeper understanding of electrical synapse plasticity in invertebrates is needed to provide further insights into the functional differences and commonalities of connexin- and innexin-based electrical synapses. If the two types did indeed use shared key mechanisms, then it will be more mysterious than ever why the vertebrates evolved a new gap junction protein to so completely replace the gap junction function of the innexins.

## Methods

### Leech care

All experiments were performed with adult leeches (*Hirudo medicinalis*) from ANIMALPHARMA GmbH (Weismain, Germany). Leeches were maintained at 18 °C in 25 l water tanks.

### Preparation of segmental ganglia

All experiments were done on electrically coupled Retzius (R) neurons from midbody ganglia (ganglia 7 to 16). Before dissection, leeches were anesthetized on ice cooled water for at least 10 min. Segmental ganglia were dissected and removed from the animal as previously described^69^ and pinned, ventral side up to a superfusion chamber coated with Sylgard (Dow Corning). Dissection was carried out in leech Ringer solution composed of (in mM): NaCl, 115; KCl, 4; CaCl_2_, 1.8; MgCl_2_, 1.5; glucose, 10; tris-(hydroxymethyl)-aminomethane (Tris) maleate, 4.6; Tris base, 5.4 (all Sigma), buffered to pH 7.4. For pharmacological experiments, the ventral glial sheath covering the ganglia was opened with a fine microscissor to ensure the direct exposure of the R cells to the drugs in the external solution.

### Electrophysiology

In all electrophysiological measurements we used the Ringer solution described above as external solution at room temperature (22 ± 2 °C). Intracellular somatic recordings and voltage clamping of the R cell pairs were performed by using two sharp glass microelectrodes (15–30 MΩ), which were pulled from borosilicate glass (GB100TF-10, Science Products) on a P-97 puller (Sutter Instrument) and filled with 3 M potassium acetate. The R cells could be unequivocally identified by their size and position within the ganglia (Fig. S2b). The microelectrodes were connected to two coupled discontinuous single electrode voltage-clamp (dSEVC) amplifiers (SEC-05X, npi electronic). Synchronizing two dSEVC amplifiers allows a precise and direct measurement of gap junctional conductance, independent of series and membrane resistances^36^. Based on an equivalent circuit model of the dual-cell voltage-clamp recordings (Fig. 1b), gap junctional conductance can readily be derived. To selectively measure only the gap junctional currents (I_j_) between the two R cells, we clamped both cells at a hyperpolarizing potential of −80 mV to inhibit the contribution of chemical synapses. In the following, the stimulated R cell will be referred to as R cell 1. Then a brief (200 ms) depolarizing voltage jump from −80 mV to −60 mV was induced in R cell 1 (V_1_). The change in the current recorded from R cell 1 (ΔI_1_) was the sum of the I_j_ and the membrane currents I_M1_. Because R cell 2 was continued to be clamped at −80 mV (V_2_), alterations of the current recorded from R cell 2 (ΔI_2_) resulted only from the temporary voltage drop between the cells and was equal to −I_j_ (Fig. 1c). Each R cell pair was only used for one experiment. We used only R cell pairs with stable membrane potentials and gap junctional currents (ΔI_2_) higher than 25 pA.

### LTP induction

To induce activity-dependent changes of the strength of electrical synapses, R cell 1 was stimulated by a depolarizing current pulse (2 nA) of different durations (1, 5 or 10 min). The prolonged depolarization caused an increase in spiking activity of the presynaptic R cell 1. The postsynaptic R cell 2 continued to be voltage clamped at −80 mV. For each R cell pair, the gap junctional currents were determined every 2 s before (baseline period) and after the spiking phase (Fig. 3a). The baseline period was 20 s (experiments with 1 min induction protocol) or 60 s (experiments with and 5 and 10 min induction protocol), respectively. The gap junctional currents were determined for a fixed standard observation period of 10 min after the spiking activity. In single experiments, junctional currents were measured as long as possible until either the tip of one of the two microelectrodes slipped out of the cell or the electrode resistance increased dramatically.

To buffer intracellular calcium concentrations, the external solution was supplemented with the membrane permeable calcium chelator 1,2-bis(o-aminophenoxy)ethane-N,N,N′,N′-tetraacetic acid acetoxymethyl ester (BAPTA-AM, 50 µM, Sigma) in dimethyl sulfoxide (DMSO, Sigma) 10 min before the experiments. As a control, the solvent DMSO (1%) was added to the external solution. To test LTP induction after washout of BAPTA-AM, the preparation was washed with external solution for 15 min by a perfusion system driven by a peristaltic pump (0.5 ml/min; Reglo Digital, Ismatec).

Considering the low membrane resistance of R cells and its relatively large size, the operation of the coupled voltage clamping needs to be critically assessed. We therefore recorded the actual membrane potential of the voltage-controlled R cell 2 with an additional microelectrode connected to a bridge amplifier (BA-01M, npi electronic) to compare it with the predetermined holding potential (Fig. S1).

### Sequence analysis

Sequence alignments were performed using Clustal W and JalView software. We used NetPhos 2.0^70^ to predict putative phosphorylation sites and TMHMM 2.0^71^ to predict transmembrane domains of Innexin-1 (accession number JQ231005, Genbank) and Innexin-14 (accession number JQ231020, Genbank), respectively.

### Data acquisition and analysis

The dSEVC amplifiers were used in a master-slave configuration with the same, synchronized switching frequency (35 kHz) and the duty cycle was set to 1/4. All current and voltage recordings were low-pass filtered at 2 kHz. For a detailed description of the operational principles of dSECV amplifiers, see^36^. Hum noise (50 Hz) was eliminated by a filter (Humbug, Quest Scientific). The signals were digitally sampled with at least 2 kHz (Micro1401, Cambridge Electronic Design), monitored by an oscilloscope (TDS 2004C, Tektronix) and recorded using the Spike2 software (Cambridge Electronic Design). All stimulation protocols were generated and delivered by a stimulus generator (Master-8, AMPI).

The junctional resistance (R_j_) and conductance (g_j_) could be simply calculated from Ohm’s law (Eq. 1). The membrane potentials (V_1_ and V_2_) and ΔI_2_ were determined from the recordings (see Fig. 1c):

1 $${g}_{j}=1/{R}_{j}={\rm{\Delta }}{I}_{2}/({V}_{1}-{V}_{2})$$

where ∆I_2_ is taken as the difference in the averaged current 100 ms prior the voltage step to −60 mV and 100 ms prior the step back to −80 mV. All data are reported as mean ± standard error (SEM) unless indicated otherwise. The normal distribution of variables was tested using the Shapiro-Wilk test. A Pearson correlation was used for all correlation coefficients. All statistical tests were performed on the raw data.

## Electronic supplementary material

Supplementary Information
