# Supplementary material for: Long-term potentiation in an innexin-based electrical synapse
Source: Sci Rep. 2018 Aug 22;8:12579. doi: 10.1038/s41598-018-30966-w (PMC6105662; doi:10.1038/s41598-018-30966-w)
Supplement: Supplementary file 1 — Supplementary Information [file 41598_2018_30966_MOESM1_ESM.pdf]

Long-term potentiation in an innexin-based electrical synapse

Georg Welzel[1] & Stefan Schuster[1]

[1]Department of Animal Physiology, University of Bayreuth, 95440 Bayreuth, Germany.

Supplementary Information

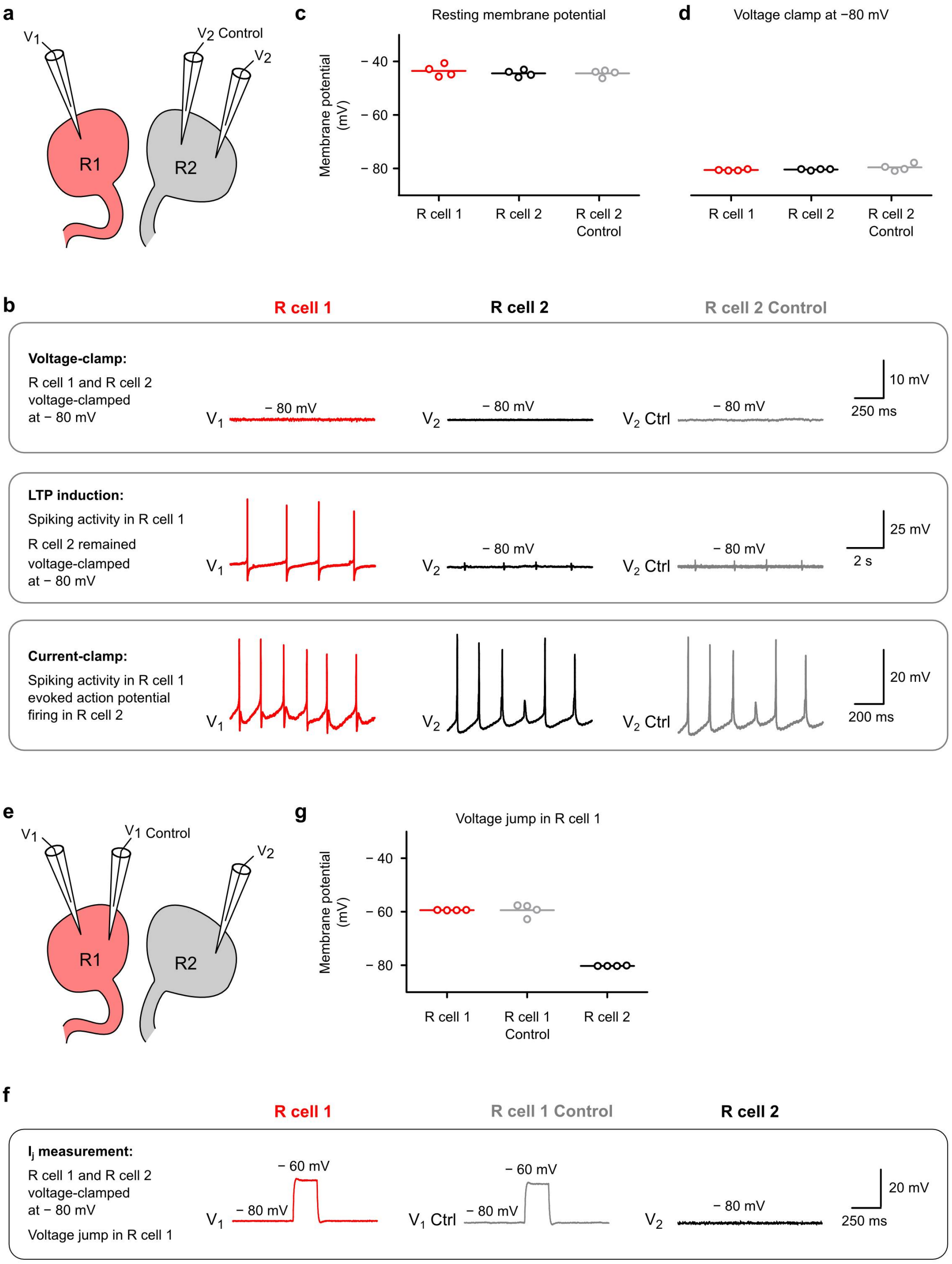

Figure S1. **The voltage of the Retzius cells is adequately controlled, even during LTP induction.** (**a**) The adequacy of the voltage-clamp protocols was confirmed by recording the actual membrane potential of the voltage controlled Retzius cell 2 with an additional independent microelectrode (V_2 Control) to compare it with the predetermined holding potential. (**b**) The voltage of the R cells was adequately voltage clamped (VC) at a holding potential of −80 mV. Even during prolonged spiking activity of R cell 1 during the LTP induction phase, the clamp circuit managed

to keep R cell 2 approximately constant at −80 mV. If R cell 2 is not voltage-clamped at −80 mV, the spiking activity induced by injecting 2 nA in R cell 1 also evoked increased spiking activity in R cell 2. (**c**) The recorded resting membrane potential and (**d**) the holding potential (−80 mV) in voltage clamp mode were confirmed by the control microelectrode. (**e**) The voltage jump from −80 mV to −60 mV in the presynaptic R cell 1 was controlled with an additional microelectrode in R cell 1 (V_1 Control). (**f** and **g**) The voltage jump to −60 mV was confirmed by the control microelectrode.

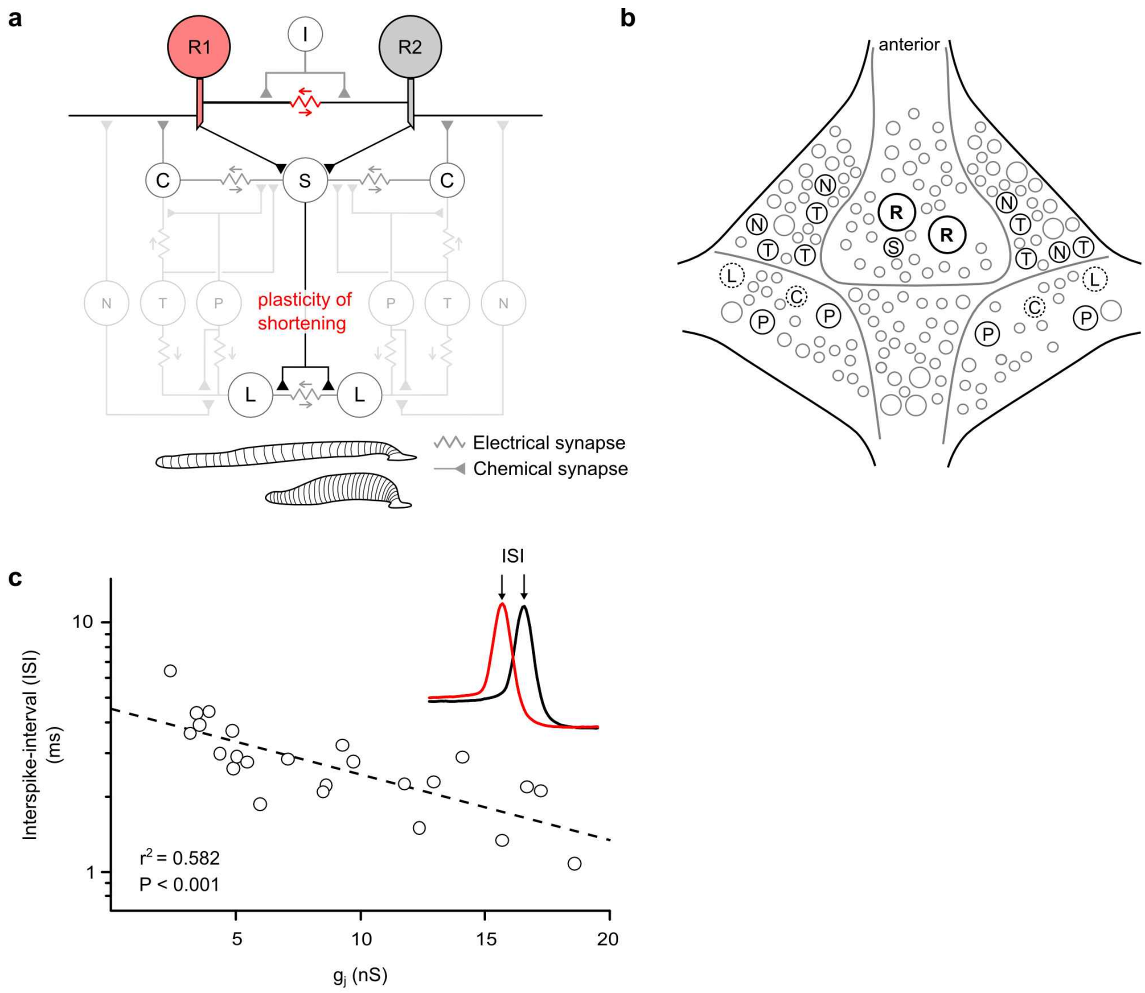

Figure S2. **Potential significance of use-dependent modulation of the electrical synapses between the Retzius cells in the leech's escape circuitry.** (**a**) The two Retzius (R) cells and their electrical synapses (red) studied here are part of a network to modulate the shortening reflex, an important escape behavior of the leech. In this network, the intersegmental S interneuron (S cell) synapses onto motor neurons (L) that control shortening via the longitudinal muscle fiber system. S cell activity, however, is regulated by excitatory serotonergic synapses from the R cells[58] that, in turn, receive chemical synaptic input from a common but yet unknown interneuron (I). In a positive feedback loop, the S cell itself excites the R cells via two coupling (C) glutamatergic interneurons[37]. (**b**) Ventral view of a schematic segmental ganglion illustrating the positions of the neurons involved in the neuronal circuit shown in (**a**). The C interneurons and L motor neurons are located on the dorsal side (dashed lines). (**c**) The interspike-interval (ISI, see inlet) decreased with an increase in gap junctional strength (r^2 = 0.582, $p < 0.001$, n = 24 R cell pairs; Pearson correlation) indicating that activity-dependent changes of g_j might cause sizeable

effects in either sensitizing or adapting the escape threshold. Data are represented as means.

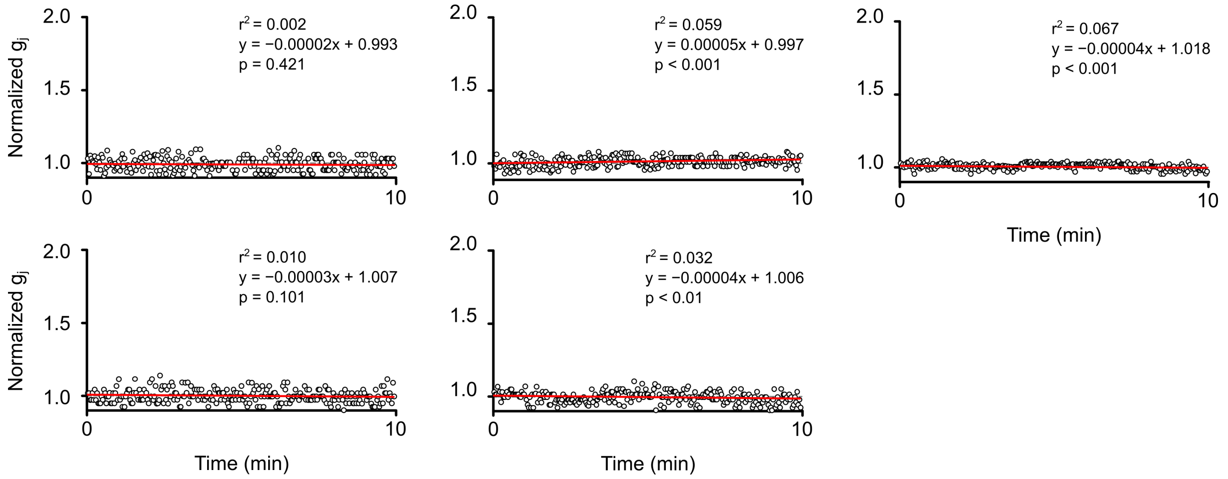

Figure S3. **Controls confirm the stability of gap junction conductance in absence of an induction phase.** Time course of normalized gap junction conductance (g_j) of all individual control recordings that were averaged in Fig. 3e ('0 min spiking') and respective linear regression lines (red; Pearson correlation; $y = g_j$ and x = time in s).

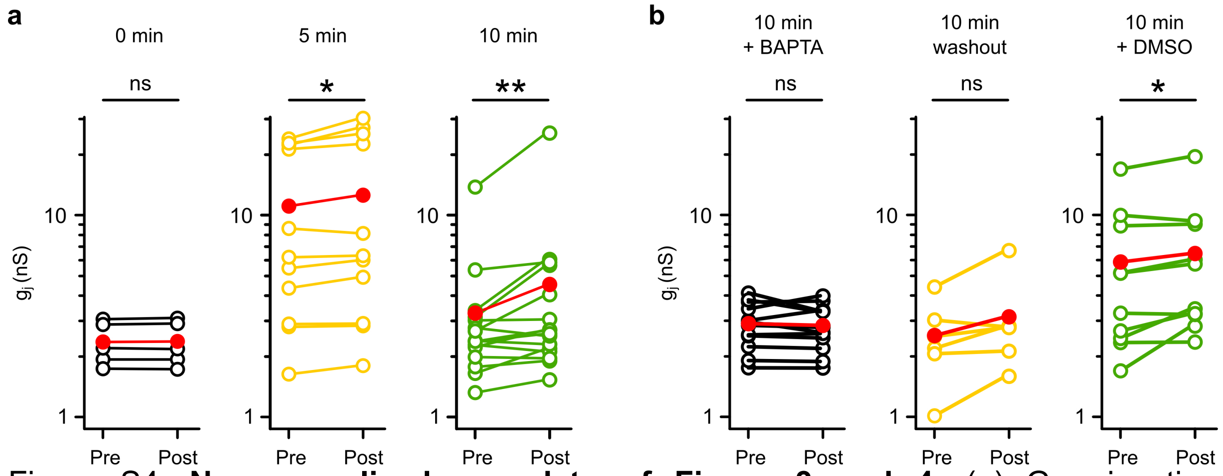

Figure S4. **Non-normalized raw data of Figure 3 and 4.** (**a**) Gap junction conductance (g_j) in nS for each individual pair of R cells. For each pair the average over time of g_j is given, first for the 1 min baseline phase (Pre) and then for the standard 10 min observation phase after spiking (Post). Duration of spiking phase, either 0 min (i.e. controls, n = 5), 5 min (n = 11) or 10 min (n = 16), as indicated. (**b**) Gap junction conductance before (Pre) and after (Post) 10 min of presynaptic spiking activity (as in (a)) in presence of BAPTA-AM (n = 13), after washout of BAPTA-AM (n = 6) and in presence of the solvent DMSO (n = 10). Mean values, calculated across the pairs, are indicated by filled red dots. ns, not significant; *, $p < 0.05$; **; $p < 0,005$, two-tailed paired Wilcoxon test.

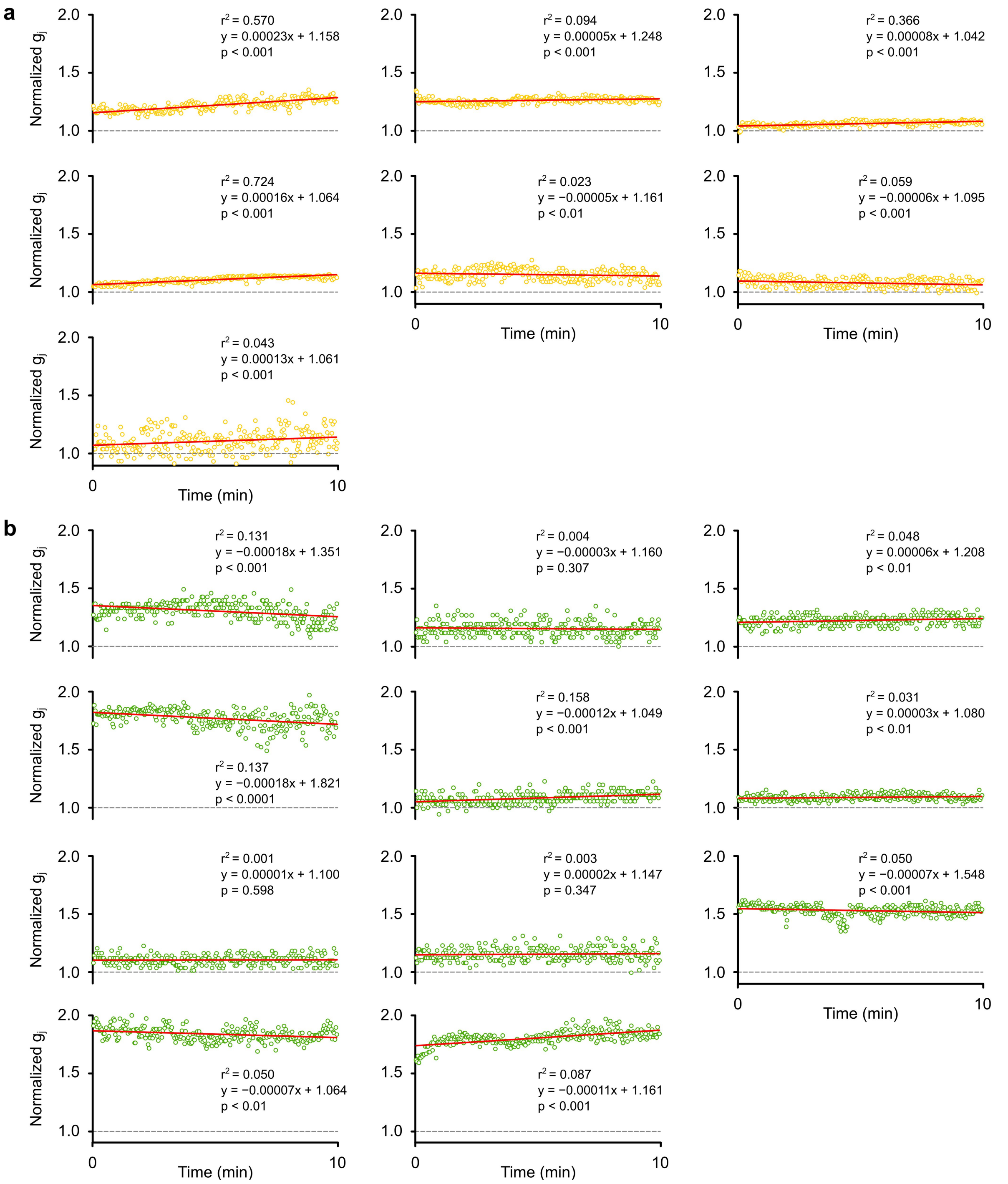

Figure S5. **Stability of LTP.** Time course of gap junction conductance (g_j), normalized to its baseline level, for all individual R cell pairs that were averaged to obtain the curves shown in Fig. 3e. (**a**) Recordings obtained after a 5 min induction phase with spiking activity. (**b**) Recordings obtained after 10 min of presynaptic spiking activity. Linear regression lines (red; Pearson correlation; y = g_j and x = time in s) highlighted to indicate that data nowhere indicate a fast decline of g_j after the end of the standard 10 min observation period. See Fig. 3 for further explanation.

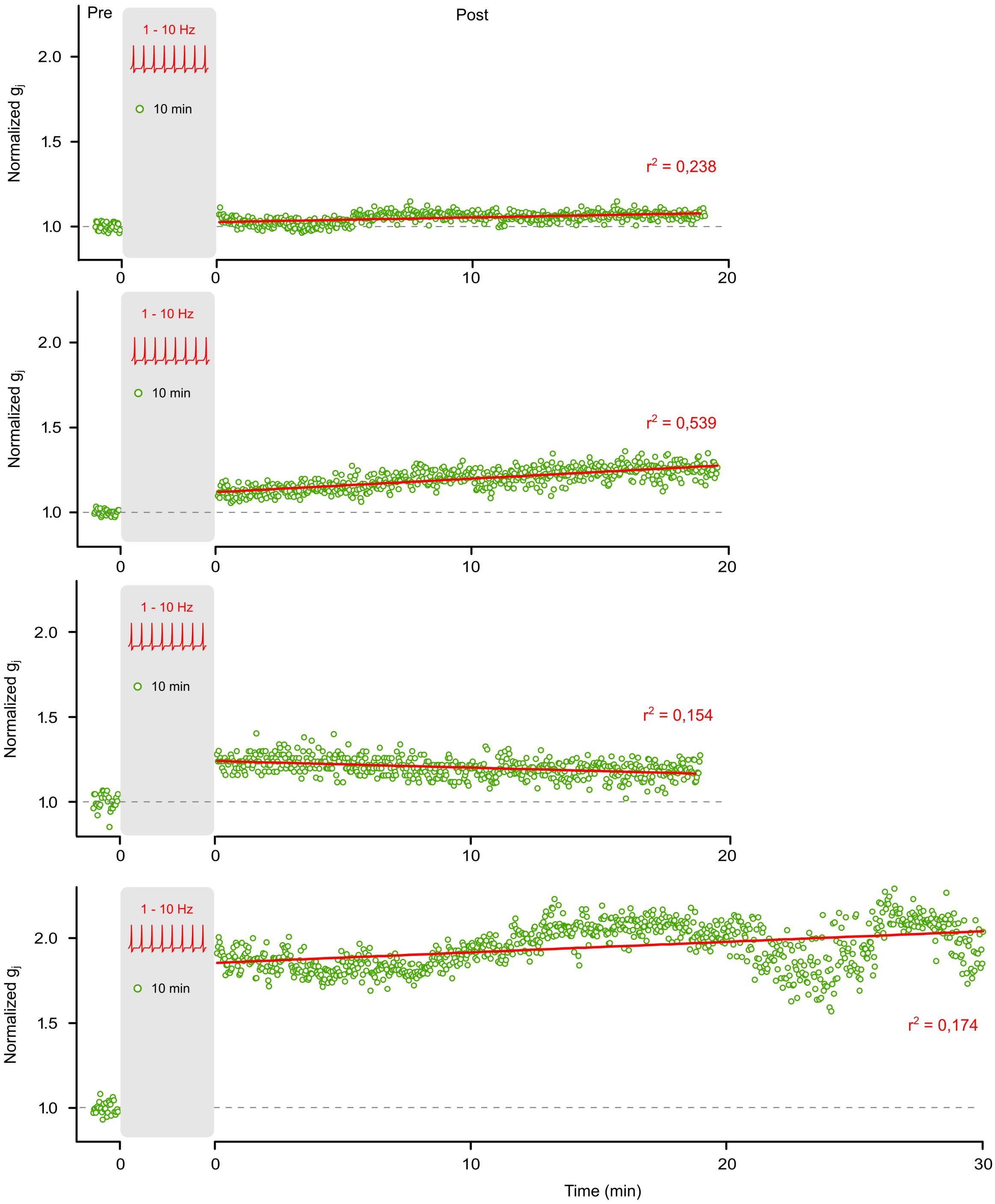

Figure S6. **Stability of long-term-potentiation extends beyond the standard 10 min recording interval.** Time course of recordings held beyond the standard interval to support analyses of Fig. S5 suggesting no decline of g_j after 10 min. Linear regression lines are shown in red (Pearson correlation). All recordings are made after 10 min of spiking.

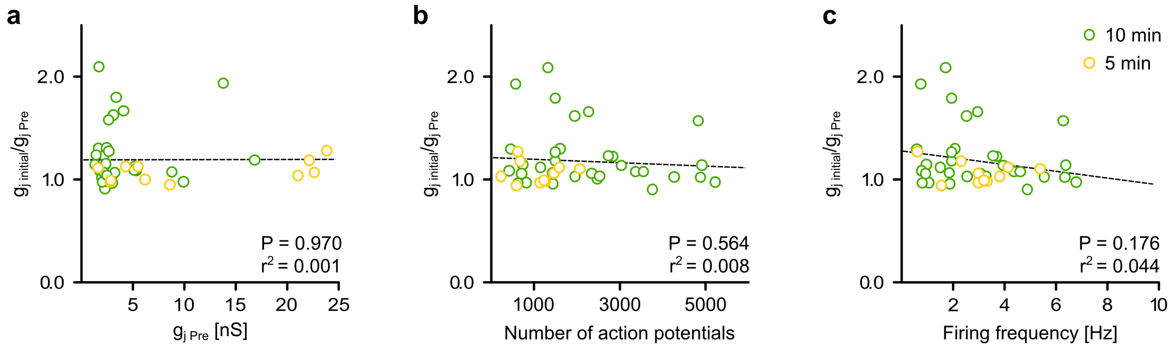

Figure S7. **Induction of LTP depended only on the duration of spiking activity.** The change of $g_{j\ initial}$ after 5 min (yellow circles) or 10 min (green circles) of presynaptic spiking activity depended neither on (**a**) the g_j before stimulation ($g_{j\ Pre}$) nor on (**b**) the total number or (**c**) frequency of action potentials fired during stimulation. Linear regression lines (dashed) are shown together with results of Pearson correlation. As in Fig. 3 $g_{j\ initial}$ is the average of the first 5 measurements of g_j after the spiking phase.
